# 2-Amino-5-bromo­pyridinium 2-carb­oxy­benzoate

**DOI:** 10.1107/S1600536810030977

**Published:** 2010-08-11

**Authors:** Ching Kheng Quah, Madhukar Hemamalini, Hoong-Kun Fun

**Affiliations:** a X-ray Crystallography Unit, School of Physics, Universiti Sains Malaysia, 11800 USM, Penang, Malaysia

## Abstract

The asymmetric unit of the title compound, C_5_H_6_BrN_2_^+^·C_8_H_5_O_4_^−^, consists of two crystallographically independent 2-amino-5-bromo­pyridinium cations (*A* and *B*) and two 2-carb­oxy­benzoate anions (*A* and *B*). Each 2-amino-5-bromo­pyridinium cation is approximately planar, with a maximum deviation of 0.047 (1) Å in cation *A* and 0.027 (1) Å in cation *B*. The 2-amino-5-bromo­pyridinium unit in cation *A* is inclined at dihedral angles of 4.9 (3) and 2.2 (3)° with the phenyl rings of the *A* and *B* 2-carb­oxy­benzoate anions, respectively. The corresponding angles for cation *B* are 3.0 (3) and 5.6 (3)°. The mol­ecular structure is stabilized by an intra­molecular O—H⋯O hydrogen bond,which generates an *S*(7) ring motif. The cations and anions are linked *via* inter­molecular N—H⋯O and C—H⋯O hydrogen bonds, generating *R*_2_^2^(8) ring motifs. In the crystal packing, mol­ecules are linked into wave-like chains along [001] *via* adjacent ring motifs. Short inter­molecular distances between the phenyl and pyridine rings [3.613 (4) and 3.641 (4) Å] indicate the existence of π–π inter­actions. The crystal structure is a non-merohedral twin with a contribution of 0.271 (3) of the minor component.

## Related literature

For applications of phthalic acid, see: Dale *et al.* (2004 ▶); Ballabh *et al.* (2005 ▶). For related structures, see: Schuckmann *et al.* (1978 ▶); Küppers (1978 ▶); Jessen & Küppers (1991 ▶); Quah *et al.* (2008 ▶, 2010*a* ▶,*b* ▶). For the stability of the temperature controller used in the data collection, see: Cosier & Glazer (1986 ▶). For bond-length data, see: Allen *et al.* (1987 ▶). For hydrogen-bond motifs, see: Bernstein *et al.* (1995 ▶).
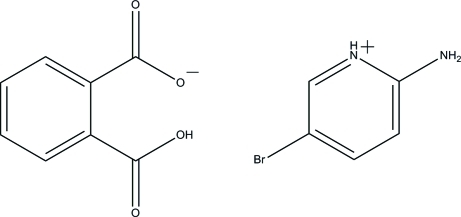


## Experimental

### 

#### Crystal data

C_5_H_6_BrN_2_^+^·C_8_H_5_O_4_^−^
*M* *_r_* = 339.15
Triclinic, 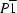

*a* = 9.0192 (4) Å
*b* = 10.2689 (5) Å
*c* = 14.4092 (6) Å
α = 82.269 (2)°
β = 83.969 (2)°
γ = 87.845 (2)°
*V* = 1314.72 (10) Å^3^
*Z* = 4
Mo *K*α radiation
μ = 3.14 mm^−1^
*T* = 100 K
0.24 × 0.20 × 0.10 mm

#### Data collection

Bruker SMART APEXII CCD area-detector diffractometer
Absorption correction: multi-scan (*SADABS*; Bruker, 2009 ▶) *T*_min_ = 0.526, *T*_max_ = 0.740
7631 measured reflections
7631 independent reflections
5583 reflections with *I* > 2σ(*I*)

#### Refinement

*R*[*F* ^2^ > 2σ(*F* ^2^)] = 0.067
*wR*(*F* ^2^) = 0.194
*S* = 1.09
7631 reflections
364 parameters
H-atom parameters constrained
Δρ_max_ = 1.14 e Å^−3^
Δρ_min_ = −1.25 e Å^−3^

### 

Data collection: *APEX2* (Bruker, 2009 ▶); cell refinement: *SAINT* (Bruker, 2009 ▶); data reduction: *SAINT*; program(s) used to solve structure: *SHELXTL* (Sheldrick, 2008 ▶); program(s) used to refine structure: *SHELXTL*; molecular graphics: *SHELXTL*; software used to prepare material for publication: *SHELXTL* and *PLATON* (Spek, 2009 ▶).

## Supplementary Material

Crystal structure: contains datablocks global, I. DOI: 10.1107/S1600536810030977/bt5311sup1.cif

Structure factors: contains datablocks I. DOI: 10.1107/S1600536810030977/bt5311Isup2.hkl

Additional supplementary materials:  crystallographic information; 3D view; checkCIF report

## Figures and Tables

**Table 1:** Hydrogen-bond geometry (Å, °)

*D*—H⋯*A*	*D*—H	H⋯*A*	*D*⋯*A*	*D*—H⋯*A*
N1*A*—H1*N*1⋯O4*A*	0.86	1.80	2.664 (7)	176
N2*A*—H2*NA*⋯O4*B*^i^	0.94	1.97	2.910 (8)	175
N2*A*—H3*NA*⋯O3*A*	0.98	1.97	2.930 (7)	167
O3*B*—H2*O*3⋯O2*B*	0.75	1.68	2.391 (6)	159
N1*B*—H2*N*1⋯O1*B*	0.92	1.82	2.647 (7)	147
N2*B*—H3*N*2⋯O1*A*^ii^	1.00	1.91	2.903 (8)	176
N2*B*—H4*N*2⋯O2*B*	0.81	2.20	2.971 (7)	160
C4*A*—H4*AA*⋯O3*B*^i^	0.93	2.44	3.219 (9)	141
C4*B*—H4*BA*⋯O2*A*^ii^	0.93	2.42	3.175 (9)	139

Symmetry codes: (i) 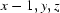; (ii) 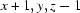.

**Fig. 1.**
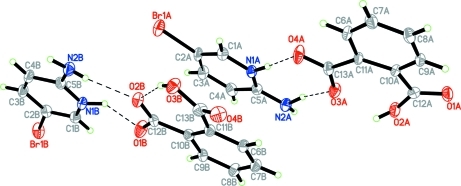
The molecular structure of the title compound showing 50% probability displacement ellipsoids for non-H atoms and the atom-numbering scheme. Intramolecular interactions are shown in dashed lines.

**Fig. 2.**
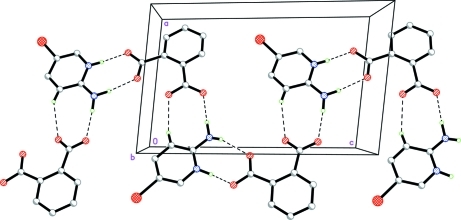
The crystal structure of the title compound viewed along the b axis. H atoms not involved in intermolecular interactions (dashed lines) have been omitted for clarity.

## supplementary crystallographic information

### Comment

Phthalic acid forms hydrogen phthalate salts with various organic and other compounds. The crystal structures of hydrogen phthalates include calcium phthalate monohydrate (Schuckmann *et al.*, 1978), lithium hydrogen phthalate monohydrate (Küppers, 1978) and tetramethylammonium hydrogen phthalate (Jessen & Küppers, 1991) have been reported in the literature. Hydrogen phthalates also form supramolecular assemblies, such as extended chains, ribbons and three-dimensional networks (Dale *et al.*, 2004; Ballabh *et al.*, 2005). In this paper, the hydrogen-bonding patterns of 2-amino-5-bromopyridinium hydrogenphthalate, (I), are discussed.

The asymmetric unit of the title compound consists of two crystallographically independent 2-amino-5-bromopyridinium cations (*A* and *B*) and two 2-carboxybenzoate anions (*A* and *B*). The bond lengths (Allen *et al.*, 1987) and angles in the title compound (Fig. 1) are within normal ranges and comparable with the related structures (Quah *et al.*, 2008, 2010*a*, *b*). Each 2-amino-5-bromopyridinium cation is approximately planar, with a maximum deviation of 0.047 (1) Å for atom Br1A in cation *A* and 0.027 (1) Å for atom Br1B in cation *B*. The 2-amino-5-bromopyridinium in cation *A* is inclined at dihedral angles of 4.9 (3) and 2.2 (3)° with the C6A—C11A and C6B—C11B phenyl rings, respectively. The correspondence angles for cation *B* are 3.0 (3) and 5.6 (3)°. The molecular structure is stabilized by an intramolecular O3B—H2O3···O2B hydrogen bond which generates an *S*(7) ring motif (Bernstein *et al.*, 1995).

The cations and anions are linked *via* intermolecular N–H···O and C–H···O hydrogen bonds (Table 1), generating *R*_2_^2^(8) ring motifs. In the crystal packing (Fig. 2), the molecules are linked into one-dimensional wave-like chains along [001] *via* adjacent ring motifs. The crystal packing is further consolidated by π-π stacking interactions between the centroids of C6A—C11A (*Cg*1), N1B/C1B—C5B (*Cg*2) rings and C6B—C11B (*Cg*3), N1A/C1A—C5A (*Cg*4) rings, with *Cg*1···*Cg*2^iii^ and *Cg*3···*Cg*4 distances of 3.613 (4) and 3.641 (4) Å, respectively [symmetry code: (iii) *x*, *y*, 1 + *z*]

### Experimental

A hot methanol solution (20 ml) of 2-amino-5-bromopyridine (86 mg, Aldrich) and phthalic acid (83 mg, Merck) was mixed and warmed over a magnetic stirrer hotplate for a few minutes. The resulting solution was allowed to cool slowly at room temperature and crystals of the title compound appeared after a few days.

### Refinement

O– and N– bound H atoms were located in a difference Fourier map and refined using a riding model with O–H = 0.7471–0.8532 Å and N–H = 0.8108–0.9952 Å]. The rest of the hydrogen atoms were positioned geometrically and refined using a riding model with C—H = 0.93 Å and *U*_iso_(H) = 1.2 *U*_eq_(C). The crystal structure is a non-merohedral twin, a contribution of  0.271 (3) of the minor component. The twin law is (-1 0 0 / 0 -1 0 /-0.320   -0.367    1).

### Crystal data

**Table:**

C_5_H_6_BrN_2_^+^·C_8_H_5_O_4_^−^	*Z* = 4
*M**_r_* = 339.15	*F*(000) = 680
Triclinic, *P*1	*D*_x_ = 1.713 Mg m^−^^3^
Hall symbol: -P 1	Mo *K*α radiation, λ = 0.71073 Å
*a* = 9.0192 (4) Å	Cell parameters from 9951 reflections
*b* = 10.2689 (5) Å	θ = 2.3–27.7°
*c* = 14.4092 (6) Å	µ = 3.14 mm^−^^1^
α = 82.269 (2)°	*T* = 100 K
β = 83.969 (2)°	Block, colourless
γ = 87.845 (2)°	0.24 × 0.20 × 0.10 mm
*V* = 1314.72 (10) Å^3^	

### Data collection

**Table:**

Bruker SMART APEXII CCD area-detector diffractometer	7631 independent reflections
Radiation source: fine-focus sealed tube	5583 reflections with *I* > 2σ(*I*)
graphite	*R*_int_ = 0.000
φ and ω scans	θ_max_ = 30.0°, θ_min_ = 1.4°
Absorption correction: multi-scan (*SADABS*; Bruker, 2009)	*h* = −12→12
*T*_min_ = 0.526, *T*_max_ = 0.740	*k* = −14→14
7631 measured reflections	*l* = −6→20

### Refinement

**Table:**

Refinement on *F*^2^	Primary atom site location: structure-invariant direct methods
Least-squares matrix: full	Secondary atom site location: difference Fourier map
*R*[*F*^2^ > 2σ(*F*^2^)] = 0.067	Hydrogen site location: inferred from neighbouring sites
*wR*(*F*^2^) = 0.194	H-atom parameters constrained
*S* = 1.09	*w* = 1/[σ^2^(*F*_o_^2^) + (0.0079*P*)^2^ + 15.1445*P*] where *P* = (*F*_o_^2^ + 2*F*_c_^2^)/3
7631 reflections	(Δ/σ)_max_ < 0.001
364 parameters	Δρ_max_ = 1.14 e Å^−^^3^
0 restraints	Δρ_min_ = −1.25 e Å^−^^3^

### Special details

**Table:**

Experimental. The crystal was placed in the cold stream of an Oxford Cyrosystems Cobra open-flow nitrogen cryostat (Cosier & Glazer, 1986) operating at 100.0 (1) K.
Geometry. All e.s.d.'s (except the e.s.d. in the dihedral angle between two l.s. planes) are estimated using the full covariance matrix. The cell e.s.d.'s are taken into account individually in the estimation of e.s.d.'s in distances, angles and torsion angles; correlations between e.s.d.'s in cell parameters are only used when they are defined by crystal symmetry. An approximate (isotropic) treatment of cell e.s.d.'s is used for estimating e.s.d.'s involving l.s. planes.
Refinement. Refinement of *F*^2^ against ALL reflections. The weighted *R*-factor *wR* and goodness of fit *S* are based on *F*^2^, conventional *R*-factors *R* are based on *F*, with *F* set to zero for negative *F*^2^. The threshold expression of *F*^2^ > σ(*F*^2^) is used only for calculating *R*-factors(gt) *etc*. and is not relevant to the choice of reflections for refinement. *R*-factors based on *F*^2^ are statistically about twice as large as those based on *F*, and *R*- factors based on ALL data will be even larger.

### Fractional atomic coordinates and isotropic or equivalent isotropic displacement parameters (Å^2^)

**Table:**

	*x*	*y*	*z*	*U*_iso_*/*U*_eq_	
Br1A	0.79304 (8)	0.52547 (8)	0.47978 (5)	0.03121 (19)	
N1A	0.6074 (6)	0.6549 (5)	0.7230 (4)	0.0196 (10)	
H1N1	0.6339	0.6324	0.7790	0.023*	
N2A	0.3893 (6)	0.7645 (6)	0.7726 (4)	0.0236 (11)	
H2NA	0.2872	0.7862	0.7731	0.02 (2)*	
H3NA	0.4231	0.7281	0.8335	0.03 (2)*	
C1A	0.7009 (7)	0.6012 (7)	0.6581 (4)	0.0223 (13)	
H1AA	0.7894	0.5596	0.6749	0.027*	
C2A	0.6649 (7)	0.6083 (7)	0.5678 (4)	0.0223 (13)	
C3A	0.5324 (7)	0.6715 (7)	0.5428 (4)	0.0243 (13)	
H3AA	0.5077	0.6766	0.4813	0.029*	
C4A	0.4383 (8)	0.7262 (7)	0.6095 (4)	0.0244 (13)	
H4AA	0.3505	0.7696	0.5932	0.029*	
C5A	0.4765 (7)	0.7158 (6)	0.7033 (4)	0.0206 (12)	
H2O3	1.0538	0.7571	0.5815	0.031*	
Br1B	0.69848 (8)	0.98559 (8)	0.01879 (5)	0.03200 (19)	
N1B	0.8885 (6)	0.8524 (6)	0.2596 (4)	0.0221 (11)	
H2N1	0.8515	0.8432	0.3223	0.027*	
N2B	1.0992 (7)	0.7300 (6)	0.2998 (4)	0.0261 (12)	
H3N2	1.2048	0.7046	0.2816	0.031*	
H4N2	1.0882	0.7577	0.3502	0.031*	
C1B	0.7963 (7)	0.9101 (7)	0.1972 (4)	0.0222 (13)	
H1BA	0.7119	0.9571	0.2180	0.027*	
C2B	0.8265 (7)	0.8995 (7)	0.1045 (4)	0.0229 (13)	
C3B	0.9539 (8)	0.8290 (7)	0.0737 (5)	0.0257 (14)	
H3BA	0.9755	0.8216	0.0101	0.031*	
C4B	1.0461 (8)	0.7713 (7)	0.1368 (4)	0.0245 (13)	
H4BA	1.1305	0.7237	0.1168	0.029*	
C5B	1.0122 (7)	0.7843 (7)	0.2339 (4)	0.0222 (13)	
O1B	0.7873 (6)	0.9253 (6)	0.4244 (3)	0.0348 (13)	
O2B	0.9788 (5)	0.8164 (5)	0.4822 (3)	0.0255 (10)	
O3B	1.0802 (6)	0.7552 (5)	0.6291 (3)	0.0301 (11)	
O4B	1.0764 (6)	0.8386 (5)	0.7617 (3)	0.0291 (11)	
C10B	0.8034 (7)	0.9257 (6)	0.5868 (4)	0.0195 (12)	
C6B	0.7961 (8)	0.9415 (7)	0.7535 (4)	0.0232 (13)	
H6BA	0.8386	0.9233	0.8101	0.028*	
C7B	0.6601 (8)	1.0105 (7)	0.7525 (5)	0.0252 (13)	
H7BA	0.6122	1.0362	0.8077	0.030*	
C8B	0.5967 (8)	1.0405 (7)	0.6684 (5)	0.0253 (13)	
H8BA	0.5074	1.0884	0.6663	0.030*	
C9B	0.6676 (7)	0.9984 (7)	0.5881 (4)	0.0216 (12)	
H9BA	0.6239	1.0188	0.5321	0.026*	
C11B	0.8708 (7)	0.8986 (6)	0.6729 (4)	0.0202 (12)	
C12B	0.8597 (7)	0.8867 (6)	0.4919 (4)	0.0200 (12)	
C13B	1.0179 (7)	0.8290 (7)	0.6895 (5)	0.0233 (13)	
O1A	0.4113 (6)	0.6654 (5)	1.2511 (3)	0.0313 (11)	
O2A	0.3986 (6)	0.7420 (5)	1.1025 (3)	0.0284 (11)	
H1OA	0.4630	0.7831	1.0627	0.043*	
O3A	0.5145 (5)	0.6946 (5)	0.9528 (3)	0.0271 (10)	
O4A	0.7025 (6)	0.5820 (6)	0.8916 (3)	0.0344 (13)	
C6A	0.8161 (7)	0.5016 (7)	1.0550 (4)	0.0222 (13)	
H6AB	0.8572	0.4762	0.9981	0.027*	
C7A	0.8877 (7)	0.4632 (7)	1.1348 (5)	0.0247 (13)	
H7AB	0.9766	0.4145	1.1309	0.030*	
C8A	0.8256 (8)	0.4979 (7)	1.2215 (4)	0.0245 (13)	
H8AB	0.8736	0.4741	1.2755	0.029*	
C9A	0.6919 (7)	0.5682 (6)	1.2255 (4)	0.0219 (12)	
H9AB	0.6506	0.5902	1.2834	0.026*	
C10A	0.6164 (7)	0.6075 (6)	1.1468 (4)	0.0197 (12)	
C11A	0.6819 (7)	0.5788 (6)	1.0570 (4)	0.0190 (12)	
C12A	0.4671 (8)	0.6747 (7)	1.1692 (5)	0.0240 (13)	
C13A	0.6295 (7)	0.6207 (7)	0.9607 (4)	0.0215 (12)	

### Atomic displacement parameters (Å^2^)

**Table:**

	*U*^11^	*U*^22^	*U*^33^	*U*^12^	*U*^13^	*U*^23^
Br1A	0.0271 (4)	0.0455 (4)	0.0232 (3)	−0.0034 (3)	0.0011 (3)	−0.0148 (3)
N1A	0.022 (3)	0.024 (3)	0.013 (2)	−0.003 (2)	−0.0037 (19)	−0.0040 (19)
N2A	0.022 (3)	0.030 (3)	0.020 (3)	−0.001 (2)	−0.002 (2)	−0.006 (2)
C1A	0.020 (3)	0.026 (3)	0.021 (3)	−0.005 (2)	−0.002 (2)	−0.004 (2)
C2A	0.024 (3)	0.030 (3)	0.014 (3)	−0.008 (3)	0.003 (2)	−0.008 (2)
C3A	0.026 (3)	0.033 (4)	0.017 (3)	−0.007 (3)	−0.006 (2)	−0.008 (3)
C4A	0.023 (3)	0.030 (4)	0.020 (3)	−0.005 (3)	−0.007 (2)	0.000 (2)
C5A	0.022 (3)	0.022 (3)	0.019 (3)	−0.005 (2)	−0.003 (2)	−0.004 (2)
Br1B	0.0262 (4)	0.0485 (5)	0.0208 (3)	−0.0002 (3)	−0.0066 (3)	0.0002 (3)
N1B	0.023 (3)	0.028 (3)	0.016 (2)	0.000 (2)	0.001 (2)	−0.006 (2)
N2B	0.028 (3)	0.031 (3)	0.019 (3)	0.004 (2)	0.000 (2)	−0.004 (2)
C1B	0.021 (3)	0.024 (3)	0.022 (3)	−0.005 (2)	−0.002 (2)	−0.004 (2)
C2B	0.022 (3)	0.028 (3)	0.020 (3)	−0.005 (3)	−0.004 (2)	−0.004 (2)
C3B	0.028 (3)	0.030 (4)	0.019 (3)	−0.006 (3)	0.003 (2)	−0.009 (3)
C4B	0.026 (3)	0.029 (3)	0.018 (3)	0.000 (3)	0.003 (2)	−0.008 (2)
C5B	0.024 (3)	0.023 (3)	0.019 (3)	−0.005 (2)	0.001 (2)	−0.002 (2)
O1B	0.036 (3)	0.051 (3)	0.018 (2)	0.014 (3)	−0.007 (2)	−0.007 (2)
O2B	0.022 (2)	0.033 (3)	0.021 (2)	0.0008 (19)	0.0014 (18)	−0.0059 (19)
O3B	0.034 (3)	0.037 (3)	0.020 (2)	0.012 (2)	−0.008 (2)	−0.007 (2)
O4B	0.029 (3)	0.033 (3)	0.028 (2)	0.003 (2)	−0.014 (2)	−0.004 (2)
C10B	0.022 (3)	0.021 (3)	0.016 (3)	−0.003 (2)	−0.003 (2)	−0.001 (2)
C6B	0.033 (3)	0.024 (3)	0.014 (3)	0.000 (3)	−0.005 (2)	−0.004 (2)
C7B	0.030 (3)	0.027 (3)	0.019 (3)	−0.003 (3)	0.001 (3)	−0.008 (2)
C8B	0.023 (3)	0.030 (4)	0.024 (3)	0.004 (3)	−0.001 (2)	−0.007 (3)
C9B	0.023 (3)	0.027 (3)	0.015 (3)	−0.002 (2)	−0.001 (2)	−0.002 (2)
C11B	0.022 (3)	0.021 (3)	0.018 (3)	−0.002 (2)	−0.001 (2)	−0.004 (2)
C12B	0.020 (3)	0.023 (3)	0.016 (3)	−0.002 (2)	−0.001 (2)	0.000 (2)
C13B	0.020 (3)	0.024 (3)	0.026 (3)	0.004 (2)	−0.008 (2)	−0.004 (2)
O1A	0.035 (3)	0.033 (3)	0.021 (2)	0.008 (2)	0.008 (2)	0.002 (2)
O2A	0.030 (3)	0.035 (3)	0.018 (2)	0.009 (2)	0.0023 (18)	−0.0023 (19)
O3A	0.027 (2)	0.036 (3)	0.019 (2)	0.010 (2)	−0.0050 (18)	−0.0047 (19)
O4A	0.033 (3)	0.057 (4)	0.013 (2)	0.015 (3)	−0.0048 (19)	−0.005 (2)
C6A	0.021 (3)	0.029 (3)	0.016 (3)	0.000 (3)	0.003 (2)	−0.006 (2)
C7A	0.021 (3)	0.028 (3)	0.022 (3)	0.002 (3)	0.001 (2)	0.002 (3)
C8A	0.026 (3)	0.030 (3)	0.018 (3)	−0.003 (3)	−0.005 (2)	0.000 (2)
C9A	0.028 (3)	0.023 (3)	0.014 (3)	−0.002 (3)	0.001 (2)	−0.004 (2)
C10A	0.023 (3)	0.020 (3)	0.016 (3)	0.000 (2)	0.002 (2)	−0.005 (2)
C11A	0.021 (3)	0.022 (3)	0.014 (3)	−0.002 (2)	0.002 (2)	−0.004 (2)
C12A	0.028 (3)	0.020 (3)	0.024 (3)	0.002 (3)	0.001 (3)	−0.004 (2)
C13A	0.022 (3)	0.028 (3)	0.015 (3)	−0.004 (2)	−0.002 (2)	−0.002 (2)

### Geometric parameters (Å, °)

**Table:**

Br1A—C2A	1.891 (6)	O3B—H2O3	0.7471
N1A—C1A	1.352 (8)	O4B—C13B	1.231 (8)
N1A—C5A	1.354 (8)	C10B—C9B	1.410 (9)
N1A—H1N1	0.8651	C10B—C11B	1.428 (8)
N2A—C5A	1.343 (8)	C10B—C12B	1.508 (9)
N2A—H2NA	0.9388	C6B—C7B	1.394 (10)
N2A—H3NA	0.9814	C6B—C11B	1.396 (9)
C1A—C2A	1.366 (9)	C6B—H6BA	0.9300
C1A—H1AA	0.9300	C7B—C8B	1.385 (9)
C2A—C3A	1.396 (10)	C7B—H7BA	0.9300
C3A—C4A	1.378 (9)	C8B—C9B	1.375 (9)
C3A—H3AA	0.9300	C8B—H8BA	0.9300
C4A—C5A	1.418 (9)	C9B—H9BA	0.9300
C4A—H4AA	0.9300	C11B—C13B	1.510 (9)
Br1B—C2B	1.889 (7)	O1A—C12A	1.226 (8)
N1B—C5B	1.340 (8)	O2A—C12A	1.302 (8)
N1B—C1B	1.352 (8)	O2A—H1OA	0.8532
N1B—H2N1	0.9235	O3A—C13A	1.267 (8)
N2B—C5B	1.345 (8)	O4A—C13A	1.239 (8)
N2B—H3N2	0.9952	C6A—C7A	1.381 (9)
N2B—H4N2	0.8108	C6A—C11A	1.421 (9)
C1B—C2B	1.353 (9)	C6A—H6AB	0.9300
C1B—H1BA	0.9300	C7A—C8A	1.399 (9)
C2B—C3B	1.400 (10)	C7A—H7AB	0.9300
C3B—C4B	1.359 (10)	C8A—C9A	1.382 (9)
C3B—H3BA	0.9300	C8A—H8AB	0.9300
C4B—C5B	1.423 (9)	C9A—C10A	1.389 (9)
C4B—H4BA	0.9300	C9A—H9AB	0.9300
O1B—C12B	1.241 (8)	C10A—C11A	1.428 (8)
O2B—C12B	1.277 (8)	C10A—C12A	1.514 (9)
O3B—C13B	1.300 (8)	C11A—C13A	1.515 (8)
			
C1A—N1A—C5A	123.1 (5)	C7B—C6B—C11B	122.4 (6)
C1A—N1A—H1N1	111.1	C7B—C6B—H6BA	118.8
C5A—N1A—H1N1	124.9	C11B—C6B—H6BA	118.8
C5A—N2A—H2NA	126.7	C8B—C7B—C6B	119.4 (6)
C5A—N2A—H3NA	109.3	C8B—C7B—H7BA	120.3
H2NA—N2A—H3NA	115.7	C6B—C7B—H7BA	120.3
N1A—C1A—C2A	119.7 (6)	C9B—C8B—C7B	119.2 (6)
N1A—C1A—H1AA	120.2	C9B—C8B—H8BA	120.4
C2A—C1A—H1AA	120.2	C7B—C8B—H8BA	120.4
C1A—C2A—C3A	119.9 (6)	C8B—C9B—C10B	123.0 (6)
C1A—C2A—Br1A	118.9 (5)	C8B—C9B—H9BA	118.5
C3A—C2A—Br1A	121.1 (5)	C10B—C9B—H9BA	118.5
C4A—C3A—C2A	119.8 (6)	C6B—C11B—C10B	118.3 (6)
C4A—C3A—H3AA	120.1	C6B—C11B—C13B	113.6 (6)
C2A—C3A—H3AA	120.1	C10B—C11B—C13B	128.2 (6)
C3A—C4A—C5A	119.4 (6)	O1B—C12B—O2B	121.6 (6)
C3A—C4A—H4AA	120.3	O1B—C12B—C10B	118.0 (6)
C5A—C4A—H4AA	120.3	O2B—C12B—C10B	120.4 (6)
N2A—C5A—N1A	119.0 (6)	O4B—C13B—O3B	120.1 (6)
N2A—C5A—C4A	122.9 (6)	O4B—C13B—C11B	120.0 (6)
N1A—C5A—C4A	118.1 (6)	O3B—C13B—C11B	119.9 (6)
C5B—N1B—C1B	122.7 (6)	C12A—O2A—H1OA	108.9
C5B—N1B—H2N1	118.9	C7A—C6A—C11A	122.3 (6)
C1B—N1B—H2N1	116.7	C7A—C6A—H6AB	118.9
C5B—N2B—H3N2	120.3	C11A—C6A—H6AB	118.9
C5B—N2B—H4N2	117.1	C6A—C7A—C8A	119.7 (6)
H3N2—N2B—H4N2	112.4	C6A—C7A—H7AB	120.2
N1B—C1B—C2B	120.1 (6)	C8A—C7A—H7AB	120.2
N1B—C1B—H1BA	120.0	C9A—C8A—C7A	119.0 (6)
C2B—C1B—H1BA	120.0	C9A—C8A—H8AB	120.5
C1B—C2B—C3B	119.6 (6)	C7A—C8A—H8AB	120.5
C1B—C2B—Br1B	119.0 (5)	C8A—C9A—C10A	122.8 (6)
C3B—C2B—Br1B	121.4 (5)	C8A—C9A—H9AB	118.6
C4B—C3B—C2B	120.0 (6)	C10A—C9A—H9AB	118.6
C4B—C3B—H3BA	120.0	C9A—C10A—C11A	119.0 (6)
C2B—C3B—H3BA	120.0	C9A—C10A—C12A	113.5 (5)
C3B—C4B—C5B	119.3 (6)	C11A—C10A—C12A	127.5 (6)
C3B—C4B—H4BA	120.3	C6A—C11A—C10A	117.1 (6)
C5B—C4B—H4BA	120.3	C6A—C11A—C13A	113.9 (5)
N1B—C5B—N2B	119.5 (6)	C10A—C11A—C13A	129.0 (6)
N1B—C5B—C4B	118.2 (6)	O1A—C12A—O2A	120.2 (6)
N2B—C5B—C4B	122.2 (6)	O1A—C12A—C10A	119.2 (6)
C13B—O3B—H2O3	121.4	O2A—C12A—C10A	120.6 (6)
C9B—C10B—C11B	117.6 (6)	O4A—C13A—O3A	122.0 (6)
C9B—C10B—C12B	114.3 (5)	O4A—C13A—C11A	118.2 (6)
C11B—C10B—C12B	128.1 (6)	O3A—C13A—C11A	119.8 (5)
			
C5A—N1A—C1A—C2A	0.5 (10)	C9B—C10B—C11B—C13B	−177.0 (6)
N1A—C1A—C2A—C3A	0.3 (10)	C12B—C10B—C11B—C13B	2.7 (11)
N1A—C1A—C2A—Br1A	−177.1 (5)	C9B—C10B—C12B—O1B	2.9 (9)
C1A—C2A—C3A—C4A	−0.1 (10)	C11B—C10B—C12B—O1B	−176.8 (6)
Br1A—C2A—C3A—C4A	177.3 (5)	C9B—C10B—C12B—O2B	−177.4 (6)
C2A—C3A—C4A—C5A	−0.9 (10)	C11B—C10B—C12B—O2B	2.9 (10)
C1A—N1A—C5A—N2A	178.9 (6)	C6B—C11B—C13B—O4B	−15.4 (9)
C1A—N1A—C5A—C4A	−1.6 (9)	C10B—C11B—C13B—O4B	163.8 (7)
C3A—C4A—C5A—N2A	−178.8 (6)	C6B—C11B—C13B—O3B	162.2 (6)
C3A—C4A—C5A—N1A	1.7 (10)	C10B—C11B—C13B—O3B	−18.6 (10)
C5B—N1B—C1B—C2B	0.5 (10)	C11A—C6A—C7A—C8A	1.5 (10)
N1B—C1B—C2B—C3B	−0.2 (10)	C6A—C7A—C8A—C9A	1.2 (10)
N1B—C1B—C2B—Br1B	−178.2 (5)	C7A—C8A—C9A—C10A	−0.7 (10)
C1B—C2B—C3B—C4B	0.2 (10)	C8A—C9A—C10A—C11A	−2.5 (10)
Br1B—C2B—C3B—C4B	178.1 (5)	C8A—C9A—C10A—C12A	175.9 (6)
C2B—C3B—C4B—C5B	−0.5 (10)	C7A—C6A—C11A—C10A	−4.6 (10)
C1B—N1B—C5B—N2B	179.7 (6)	C7A—C6A—C11A—C13A	174.7 (6)
C1B—N1B—C5B—C4B	−0.7 (10)	C9A—C10A—C11A—C6A	5.0 (9)
C3B—C4B—C5B—N1B	0.7 (10)	C12A—C10A—C11A—C6A	−173.1 (6)
C3B—C4B—C5B—N2B	−179.7 (7)	C9A—C10A—C11A—C13A	−174.2 (6)
C11B—C6B—C7B—C8B	−1.2 (11)	C12A—C10A—C11A—C13A	7.7 (11)
C6B—C7B—C8B—C9B	1.7 (10)	C9A—C10A—C12A—O1A	−14.9 (9)
C7B—C8B—C9B—C10B	−0.2 (11)	C11A—C10A—C12A—O1A	163.3 (7)
C11B—C10B—C9B—C8B	−1.7 (10)	C9A—C10A—C12A—O2A	164.9 (6)
C12B—C10B—C9B—C8B	178.5 (6)	C11A—C10A—C12A—O2A	−16.9 (10)
C7B—C6B—C11B—C10B	−0.7 (10)	C6A—C11A—C13A—O4A	2.3 (9)
C7B—C6B—C11B—C13B	178.5 (6)	C10A—C11A—C13A—O4A	−178.5 (7)
C9B—C10B—C11B—C6B	2.1 (9)	C6A—C11A—C13A—O3A	−177.0 (6)
C12B—C10B—C11B—C6B	−178.2 (6)	C10A—C11A—C13A—O3A	2.2 (10)

### Hydrogen-bond geometry (Å, °)

**Table:**

*D*—H···*A*	*D*—H	H···*A*	*D*···*A*	*D*—H···*A*
N1A—H1N1···O4A	0.86	1.80	2.664 (7)	176
N2A—H2NA···O4B^i^	0.94	1.97	2.910 (8)	175
N2A—H3NA···O3A	0.98	1.97	2.930 (7)	167
O3B—H2O3···O2B	0.75	1.68	2.391 (6)	159
N1B—H2N1···O1B	0.92	1.82	2.647 (7)	147
N2B—H3N2···O1A^ii^	1.00	1.91	2.903 (8)	176
N2B—H4N2···O2B	0.81	2.20	2.971 (7)	160
C4A—H4AA···O3B^i^	0.93	2.44	3.219 (9)	141
C4B—H4BA···O2A^ii^	0.93	2.42	3.175 (9)	139

Symmetry codes: (i) *x*−1, *y*, *z*; (ii) *x*+1, *y*, *z*−1.
